# Comment on Kierlik et al. Application of Mössbauer Spectroscopy for Identification of Iron-Containing Components in Upper Silesian Topsoil Being under Industrial Anthropopressure. *Materials* 2020, *13*, 5206

**DOI:** 10.3390/ma15155214

**Published:** 2022-07-28

**Authors:** Pranaba K. Nayak

**Affiliations:** Department of High Energy Physics, Tata Institute of Fundamental Research, Mumbai 400005, India; pranaba@hotmail.com; Tel.: +91-22-2278-2367

**Keywords:** Mössbauer spectroscopy, magnetite, topsoil, magnetic separation, line width, hyperfine field

## Abstract

Kierlik et al. recently reported their study in this journal on the application of Mössbauer spectroscopy in identifying the iron-containing components in Upper Silesian topsoil as being under industrial anthropopressure. While the presented work is an excellent example of characterizing topsoil, a significant part of the study involving iron-bearing samples using ^57^Fe Mössbauer spectroscopy is erroneous. In the spectral fitting routine for the obtained Mössbauer spectra of the sample IIA (Figure 2; Table 3), the authors have erroneously fitted only a single sextet for magnetite, which is unacceptable. The line width obtained by them is also incorrectly mentioned in Figure 2, as evident from fitting D1 and D2 doublets as well as of magnetite tetrahedral and octahedral sites, making the relative abundances of individual phases meaningless. The inaccurate line width makes the authors fit the inadequate number of doublets necessary for the iron-bearing phases present in these samples, resulting in incorrect relative abundance for the other phases found in the materials, making the subsequent discussion not much useful.

For the sample IIA, XRD (Figure 1 and Table 2) confirmed the presence of a good quantity of magnetite, but the authors have fitted only one sextet for magnetite to the obtained Mössbauer spectrum, which is erroneous [1]. For stoichiometric magnetite, the hyperfine parameters from Mössbauer spectra are well-established and widely reported, with a two-sextet routine corresponding to the tetrahedral and the octahedral sites with the chemical shift (relative to α-iron) at 0.26 mm s^−1^ and 0.67 mm s^−1^ with hyperfine fields 49 T and 46 T, respectively [2]. With cubic structure, the quadrupole shift value is around zero for both sites, encouraging many to ignore it while reporting. At room temperature, two overlapping sextets are observed, one arising from ferric ions in the tetrahedral sites and the other being a time-averaged sextet, arising from ferrous and ferric ions in the octahedral sites with fast electron fluctuations between them. Between these two sextets, the one corresponding to the octahedral site possesses a relatively broader linewidth as it is composed of two B-site sextets (containing both ferrous and ferric ions) as a result of two different possible directions of the magnetic hyperfine field with respect to the local B-site EFG principal axes [3]. However, magnetite is recognized readily from its room temperature Mössbauer spectrum of the tetrahedral site, making two-sextet fitting adequate for characterization purposes. Consequently, any fitting routine comprising only a single sextet, ignoring the sextet for the tetrahedral site of magnetite, is not acceptable. Moreover, for this sample (IIA), the obtained isomer shift value for the magnetite octahedral site is significantly low (0.5 mm s^−1^ indicates only the ferric component), which overlooks the presence of the ferrous component.

In observing Figure 2, it can be understood from the spectra for the samples (IA, IIA, IB and IIB) that there is a significant difference in line width of doublets D1 and D2, which were reported to be within the range of 0.35–0.4 mm s^−1^ (Table 2 caption). Similar observation can be made comparing the line width of the tetrahedral sextet to the octahedral sextet line width for the sample IA. The large line width indicates that an inadequate number of phases were considered for the purpose, as the authors also should have realized, since they suspect maghemite, ferrihydrite and pyrite to be present (first paragraph; page 7). Hence, incorrectly fitted iron-bearing phases in these samples made the quantification and subsequent discussions, not much meaningful, considered the best feature in Mössbauer spectral analysis. We do not understand why the authors have not reported individual line width that is warranted, which would provide a concrete idea about the presence or absence of any phase or component on a standalone basis.

In summary, authors need to assign proper iron-bearing phases to the so delicately collected Mössbauer spectra before the re-analysis and subsequent presentation of their results appended in Figures (Figure 2) and Tables (Tables 3 and 4). This it will make the efforts worthy, and a valuable reference for future researchers.
